# Longitudinal assessment of glycaemic management and neurophysiological outcomes in paediatric type 1 diabetes in Switzerland: the KIND cohort profile

**DOI:** 10.1136/bmjopen-2026-116918

**Published:** 2026-11-11

**Authors:** Marc Robin Gruener, Erin West, Ute Muhitira, Katrin Heldt, Sarah Oberhauser, Dagmar l’Allemand-Jander, Philip Julian Broser, on behalf of the KIND Study Group, Philip J. Broser

**Affiliations:** 1 Neurophysiology, Ostschweizer Kinderspital, St. Gallen, Switzerland; 2 Clinical Trials Unit, HOCH Health Ostschweiz, St. Gallen, Switzerland; 3 Endocrinology & Diabetology, Ostschweizer Kinderspital, St. Gallen, Switzerland; 4 Metabolic Centre St. Gallen, St. Gallen, Switzerland; 5 University of Basel Faculty of Medicine, Basel, Switzerland

**Keywords:** Diabetic neuropathy, Paediatric neurology, Paediatric endocrinology, ULTRASONOGRAPHY, Diabetes Mellitus, Type 1, Nerve Conduction Studies

## Abstract

**Abstract:**

**Purpose:**

The KINder mit Diabetes cohort investigates diabetic peripheral neuropathy (DPN) in paediatric type 1 diabetes (T1D). Current guidelines recommend DPN screening at puberty or from 11 years and 2–5 years after T1D diagnosis, yet subclinical neurophysiological changes occur within the first 2 years. The cohort examines: (1) longitudinal associations between glycaemic metrics (haemoglobin A1C and continuous glucose monitoring-derived variability metrics) and peripheral nerve function and structure; (2) comparative predictive value of different variability metrics; (3) developmental trajectories of nerve maturation in T1D versus controls; (4) effects of residual beta-cell function on neuropathy progression and (5) how early signs of DPN differ between patients with multiple daily injections and continuous subcutaneous insulin infusion therapy.

**Participants:**

This prospective cohort, initiated in June 2019, continuously enrols children and young adults with T1D (≤21 years) at two Swiss centres. The care-embedded design integrates research into quarterly diabetes care and annual comprehensive assessments. So far, 179 T1D cohort participants (median age: 11.3; IQR: (7.6; 14.2) years; 49.2% female) and 123 healthy controls (10.8 (7.3; 14.6) years; 56.1% female) were recruited. Controls for neurophysiological examinations comprise measurements from the healthy, contralateral side of children with limb injuries in the surgical outpatient clinic of the Children’s Hospital of Eastern Switzerland (OKS). Multimodal assessments comprise Nerve Conduction Studies (peroneal, tibial, median motor and sensory) and high-resolution ultrasound, with development-adjusted analyses distinguishing diabetes effects from normal growth.

**Findings to date:**

Cross-sectional analysis showed reduced nerve conduction velocities across all nerves, particularly peroneal in T1D patients (n=53) compared to healthy controls (n=50). Peroneal height-adjusted nerve conduction velocity (dNCV) correlated negatively with glucose variability (SD: r=−0.45, p=0.009) and HbA1c (r=−0.27, p=0.049). During the first five years, dNCV correlated negatively with diabetes duration (r=−0.41, p=0.004), independent of glycaemic control. The cross-sectional area of the median nerve increased on average by 0.217 mm² per 1% HbA1c (p=0.004) and was already detectable with diabetes duration <2 years. Longitudinal analysis (n=45, median interval 17.9 (16.1; 25.8) months) demonstrated that HbA1c changes predicted dNCV changes (β=−0.59, p=0.014), and in some patients, early impairment was reversible with improved glycaemic control.

**Future plans:**

A planned 2026 extension of this continuously recruited and prospectively followed cohort will integrate physical activity measures (Swiss National Science Foundation Grant 10006264). Future analyses will compare glycaemic variability metrics as predictors of functional and structural nerve changes, investigate their temporal relationships as well as influencing factors, and examine residual beta-cell function effects across developmental stages.

**STRENGTHS AND LIMITATIONS OF THIS STUDY**

- Longitudinal clinical and neurophysiological investigation from type 1 diabetes diagnosis through adolescence captures peripheral neuropathy development compared with age-matched healthy controls.
- Integration of nerve conduction studies with high-resolution ultrasound provides comprehensive functional and structural assessment using non-invasive and development-adjusted analyses.
- Continuous glucose monitoring and quarterly HbA1c enable precise quantification of glycaemic control and comparison of multiple variability metrics as neuropathy predictors.
- Two-centre regional study limits geographic and ethnic diversity.
- A broad variety of continuous glucose monitoring devices across manufacturers complicate metric standardisation despite harmonisation efforts.
- Normative reference values derive from the clinically unaffected contralateral limb of children with a unilateral injury rather than from healthy volunteers, though these values agree with published paediatric norms.

## Introduction

 Diabetic peripheral neuropathy (DPN) is the most common microvascular complication of diabetes, affecting nearly half of all patients during their lifetime.^1–4^ In children and adolescents with type 1 diabetes (T1D), DPN manifests as dysfunction of peripheral nerves, typically in a ‘stocking-and-glove’ distribution, and is associated with impaired balance, sensory loss and pain.^5^ Prevalence rates in paediatric populations range from 7% to over 57%, depending on diagnostic criteria and patient characteristics.^6^ Longitudinal studies demonstrate that DPN begins early and progresses throughout childhood: Hyllienmark *et al*^7^ reported a 15% incidence of clinical neuropathy within 13 years, while Hajas *et al*^8^ observed subclinical DPN prevalence increasing from 24 to 63% over ten years. Despite this early onset and progressive nature, current International Society for Pediatric and Adolescent Diabetes (ISPAD) guidelines recommend screening at puberty or from 11 years of age and 2 to 5 years postdiagnosis of T1D. However, recent evidence demonstrates that subclinical changes are detectable within the first 2 years following diagnosis of T1D,^7 9 10^ highlighting the need for early detection methods and better understanding of modifiable risk factors before irreversible nerve damage occurs.

Several fundamental questions remain unanswered regarding DPN pathogenesis and progression in children and adolescents. First, while haemoglobin A1C (HbA1c) predicts long-term complication risk,^11 12^ glycaemic variability (GV) has emerged as a potentially independent contributor to neuronal injury.^9^ Rapid glucose oscillations, common in paediatric T1D, may induce oxidative stress and pro-inflammatory signalling,^13–15^ but it remains unclear how well GV metrics (SD, coefficient of variation, time-in-range or more complex indices)^16–19^ predict neurophysiological changes over time. The widespread use of continuous glucose monitoring (CGM) enables detailed GV assessment, yet device heterogeneity across manufacturers complicates metric standardisation and comparability.^20^ Second, distinguishing diabetes-related nerve impairment from normal developmental changes poses a unique paediatric challenge. Nerve conduction velocity (NCV) increases dramatically from 20 m/s at birth to 45 m/s by age 2, with maturation continuing into puberty, while nerve cross-sectional area (CSA) expands proportionally with body growth.^21^ Whether age at diabetes diagnosis influences neuropathy trajectories through interactions with nervous system development remains unknown. Third, residual beta-cell function during the remission phase may be protective against complications,^22^ and preliminary observations suggest that peroneal height-adjusted NCV (dNCV; defined as the difference between measured and, given a patient’s height, expected NCV) declines independently of glycaemic control during remission phase cessation.^9^ Whether C-peptide levels modify neuropathy progression requires longitudinal investigation. Finally, standard clinical screening tools (monofilament testing, vibration perception)^23^ have overall poor sensitivity in children,^24 25^ while more sensitive functional (nerve conduction studies, NCS) and structural (high-resolution ultrasound) assessments can detect subclinical changes,^9 24 25^ but how these modalities relate to each other temporally remains poorly characterised.

Addressing these knowledge gaps requires a cohort design that captures both disease progression and developmental maturation over extended periods. The KINder mit Diabetes (KIND) cohort employs continuous enrolment from T1D diagnosis onward, enabling investigation of neuropathy from its earliest stages through adolescence and young adulthood. This approach spans the full spectrum of diabetes duration and developmental stages. Integration into routine clinical care, quarterly visits with annual comprehensive assessments, maximises retention and minimises patient and family burden.

A control group of children undergoing neurophysiological evaluation for unilateral fractures provides normative developmental reference values, taken from the unaffected limb, for these adjustments. This comprehensive, care-embedded approach enables investigation of how glycaemic patterns interact with developmental stage and residual beta-cell function to influence peripheral nerve structure and function.

The KIND cohort investigates five interrelated objectives: first, to characterise longitudinal associations between glycaemic control metrics (HbA1c and GV indices) and peripheral nerve function and structure in children and adolescents with T1D; second, to compare the predictive value of different GV metrics for neurophysiological outcomes; third, to delineate developmental trajectories of peripheral nerve maturation in T1D compared with controls, accounting for age, pubertal stage and diabetes duration; and fourth, to assess the influence of residual beta-cell function (C-peptide) on neuropathy progression, particularly during the remission phase. Fifth, the study examines how glycaemic control and early signs of neuropathy differ between multiple daily injection (MDI) and continuous subcutaneous insulin infusion (CSII) therapy. By evaluating these objectives, the cohort is expected to identify modifiable risk factors for early intervention and provide information for optimising strategies of paediatric diabetes treatment.

## Cohort description

### Study design and setting

The KIND longitudinal cohort was started in June 2019. This ongoing prospective observational cohort study is conducted at two centres in Eastern Switzerland: the Children’s Hospital of Eastern Switzerland (OKS) in St. Gallen and the St. Gallen Metabolic Centre (SGMC).

The cohort is designed as a ‘care-embedded’ study, integrating research data collection into the routine clinical care pathway for paediatric patients with T1D. This approach maximises participant retention while minimising burden on families.

Both centres follow the most current ISPAD guidelines 2022/2024 for diabetes management. T1D diagnosis is confirmed according to ISPAD clinical and laboratory criteria.^26 27^ Most patients with T1D at both centres use CSII with sensor augmentation or hybrid closed-loop systems, with CGM as standard practice also in children using an MDI regimen, consistent with ISPAD technology guidelines.^28 29^ Specialised neurophysiological evaluations include NCS and high-resolution nerve ultrasound, assessments that extend beyond routine clinical practice according to ISPAD guidelines.

### Recruitment and eligibility

The KIND cohort enrols individuals with T1D age 21 years and younger continuously at T1D diagnosis and during routine clinical visits. This creates a diverse cohort spanning the full spectrum of diabetes duration from newly diagnosed to long-standing disease in children and adolescents, enabling investigation of how the physiological developmental stage at T1D diagnosis influences DPN pathogenesis. Eligibility criteria apply at two levels. Both arms require an age of 21 years or younger at enrolment, written informed consent from the participant and/or their legal guardians, and the absence of acute illness and of any condition known to affect peripheral nerve function or structure: premature birth (<32 gestational weeks), inherited or acquired neuropathy of any cause, personal or family history of hereditary neurological disease, treatment with neurotoxic agents including chemotherapy, vitamin B_12_ deficiency, untreated hypothyroidism, chronic kidney disease, or chronic inflammatory or rheumatological disease. In addition, the T1D arm requires a diagnosis confirmed according to ISPAD criteria, and the control arm a unilateral upper-limb injury with a clinically unaffected contralateral side, excluding diabetes, impaired glucose tolerance, and suspected injury to the brachial plexus or to the nerves of the reference side. Eligibility was ascertained from the clinical history and the clinical record.

The neurophysiology department at OKS routinely evaluates patients with unilateral fractures and suspected nerve trauma. As standard care, these patients undergo peripheral neuropathy screening (see Neurophysiology assessment) of the injured and uninjured extremity, typically 10 days after the trauma. Consecutively presenting patients meeting the criteria above are invited to participate. The measurements taken from the uninjured side serve as the control for neurophysiological and ultrasonographic analyses.^9^ We applied this same screening protocol to our T1D cohort as described below.

### Follow-up timeline

The KIND cohort is based on a longitudinal follow-up design that uses the established framework of clinical care at both participating centres (figure 1), ensuring a high retention rate, comprehensive data collection and minimised patient burden.

**Figure 1:**
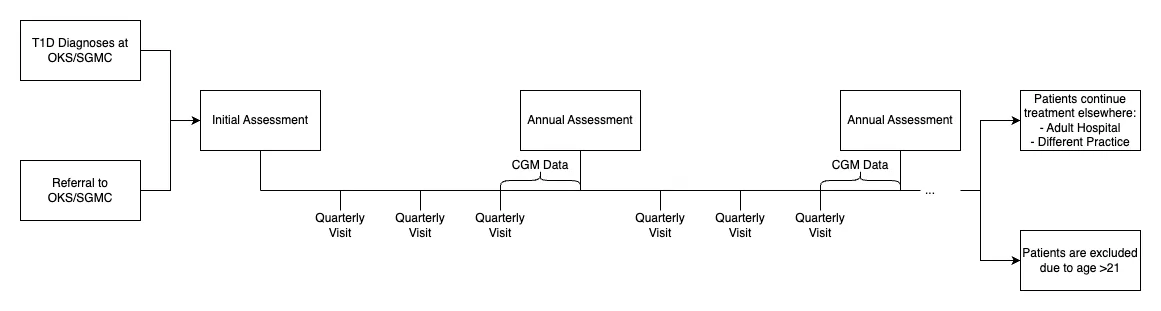
Overview of the longitudinal study design. CGM, Continuous Glucose Monitoring; OKS, Children’s Hospital of Eastern Switzerland; SGMC, St. Gallen Metabolic Centre; T1D, Type 1 Diabetes.

Following T1D diagnosis or initial enrolment, patients undergo a comprehensive baseline assessment that establishes detailed clinical, endocrinological and specialised neurophysiological profiles including extensive laboratory testing. Subsequently, the study follows standard paediatric diabetes care protocols as outlined by ISPAD guidelines^30^ and previously described,^9^ with quarterly visits as well as annual comprehensive assessments which include diabetes management assessment and the research-specific data collection.^18^ The anticipated primary reasons for study discontinuation are family relocation or transition to adult care.

## Data collection

Both participating centres use identical assessment protocols and comparable equipment, ensuring consistency in longitudinal measurements and enabling valid data pooling across sites. For neurophysiological assessments, patients registered at SGMC are referred to OKS, where specialised equipment and trained personnel are available to conduct NCS and high-resolution nerve ultrasound.

### Clinical measures

Standard growth parameters (height, weight, waist circumference) are measured using calibrated equipment following established protocols. These measurements enable calculation of derived indices including BMI, waist-to-height ratio, and body surface area (BSA; Du Bois formula^31^ expressed as sex-adjusted and age-adjusted variables (z-scores, referring to Swiss/WHO reference data,^32^ if appropriate).

### Diabetes care

Assessments for diabetes care are presented in table 1 and based on standardised protocols in accordance with ISPAD 2022 Clinical Practice Consensus Guidelines, incorporating 2024 updates.^33 34^ Glycaemic characterisation combines quarterly HbA1c measurements with CGM data focused on 90-day windows aligned with the HbA1c integration period of 2 to 3 months,^35^ enabling calculation and comparison of multiple GV metrics to determine their relative predictive value for neurophysiological outcomes. The comprehensive evaluation of the cohort characterises diabetes management, growth patterns and clinical status essential for investigating study aims.

#### Physical examination

Standardised examination includes blood pressure measurements, systematic assessment of insulin injection/infusion sites for lipohypertrophy, and annual clinical neuropathy screening using monofilament testing and vibration perception assessment.

#### Diabetes management assessment

Comprehensive documentation (table 1) includes insulin delivery method (CSII vs MDI), device specifications (manufacturer, standalone/sensor-augmented/hybrid closed-loop systems), CGM device details (overview in online supplemental table 1), total daily insulin dose, insulin-to-carbohydrate ratios and diabetes-related symptoms.

**Table 1:** Data collection timeline and measurements

Time point	Quarterly visits	Additional during annual assessment
Clinical measures	Height, weight, blood pressure, heart rate	+Waist circumference, pubertal stage
Diabetes care	Device/therapy review, total daily insulin dose, glucose monitoring and injection site examination	Nothing additional to Quarterly Visits
CGM data	Data export and review*	Nothing additional to Quarterly Visits
Laboratory	HbA1c, random capillary blood glucose	+Random C-peptide, creatinine, lipid profile, electrolytes, screening for thyroid and coeliac disease, albumin-creatinine ratio in urine
Neurophysiology	–	+NCS, high-resolution nerve ultrasound, clinical assessment

* CGM data collection occurs continuously, and data are exported and reviewed during quarterly visits.

CGM, Continuous Glucose Monitoring; HbA1c, Haemoglobin A1c; NCS, Nerve Conduction Studies.

### CGM data collection

#### Data sources and collection

CGM data are collected from systems commonly used in clinical practice: FreeStyle Libre 1/2/2+/3/3+ (Abbott Diabetes Care, Alameda, California, USA), Dexcom G6/G7 (Dexcom, San Diego, California, USA), and Medtronic Guardian 3/4/Simplera (Medtronic MiniMed, Northridge, California, USA). Data are accessed through manufacturer-specific platforms or the integrated diabetes management systems Glooko (Glooko AB, Gothenburg, Sweden), Tidepool (Tidepool Project, Palo Alto, California, USA) and Diabass (mediaspects Beratungsgesellschaft für neue Medien, Balingen, Germany).

CGM data were collected as comprehensively as possible, with particular focus on the 90-day period preceding each neurophysiological assessment. This 90-day window aligns with the HbA1c integration period. When 90-day data are unavailable, shorter periods (14–90 days) can be used, given the high correlation between 14-day and long-term CGM metrics.^36^

Routine manual calibration of CGMs is not required unless specified by the device manufacturer (eg, Medtronic) or indicated to parents/patients by suspected CGM inaccuracy.

#### Data harmonisation challenge

Patients at OKS and SGMC use a variety of CGM devices (table 2). Recent publications have highlighted performance differences and metric comparability issues across CGM manufacturers and devices,^20 37 38^ prompting standardised data preprocessing.

**Table 2:** Diabetes variables of the KIND cohort at baseline

	Median	(Q1; Q3)	n
**Basics**			
Age at diagnosis (year)	7.9	(4.2; 11.3)	179
Diabetes duration (year)	2.0	(0.0; 4.8)	179
pH at diagnosis	7.3	(7.2; 7.4)	162
Bicarbonate at diagnosis (mmol/L)	12.7	(6.4; 19.8)	152
**Laboratory**			
HbA1C (%)	8.1	(7.2; 10.8)	172
C-peptide (pmol/L)	53.0	(0.0; 169.0)	144
Sodium (mmol/L)	138.9	(137.0; 140.0)	117
Potassium (mmol/L)	4.3	(4.0; 4.5)	115
Chloride (mmol/L)	103.0	(101.0; 105.9)	113
Ionised calcium (mmol/L)	1.2	(1.2; 1.2)	98

	**Share**	**n**
**DKA at diagnosis** *		
No DKA (%)	30.7	55
Mild (%)	22.3	40
Moderate (%)	17.9	32
Severe (%)	19.6	35
Missing (%)	9.5	17
**CGM** **†**		
Patients with ≥70% CGM data available (%)‡	34.1	61
CGM devices§		
Abbott (%)	21.2	38
Dexcom (%)	24.0	43
Medtronic (%)	15.1	27
No CGM data (%)‡	40.8	73

Initial CGM characteristics are reported in online supplemental table 4.

* Diabetic ketoacidosis (DKA) at diagnosis is classified according to ISPAD 2022; where only one of the two markers was available, classification was based on that marker alone.

† CGM related data are for the 90 days preceding baseline NCS.

‡ Over 30% (n=57) of the cohort were enrolled within 3 months of diagnosis, before CGM therapy is established, reasons for ‘no CGM data’ include newly diagnosed patients, patients not wearing a CGM as part of their therapy, or no access to historical CGM data.

§ Two patients have used CGM devices by two different manufacturers; percentages are of all 179 participants and therefore sum to slightly more than 100%.

CGM, Continuous Glucose Monitoring; HbA1c, Haemoglobin A1c; ISPAD, International Society for Pediatric and Adolescent Diabetes; KIND, KINder mit Diabetes; NCS, Nerve Conduction Studies.

If necessary, CGM values are converted from mg/dl to mmol/L (conversion factor: 0.0555) and timestamps are standardised to Coordinated Universal Time before database storage.

Future analyses will require additional harmonisation to address device-specific differences in measurement frequencies (ranging from 1 to 15-minute intervals) and out-of-range value handling (online supplemental table 1). Manufacturers use different measurement ranges and coding approaches. Preprocessing to date resampled all recordings to a common 15-minute grid by forward-fill with a limit of one interval and treated out-of-range values as missing (online supplemental table 4) or set to the sensors’ upper and lower limits, pending analysis-specific handling. Temporal alignment, out-of-range coding and minimum data sufficiency are decided per analysis rather than fixed for the cohort, since velocity-based metrics such as Mean Absolute Glucose, Glucose Variability Percentage and the Glucose Fluctuation Moment Index are more sensitive to sampling frequency than mean glucose, SD or time-in-range. Device manufacturer and model are carried as covariates, or as the basis of single-device sensitivity analyses, wherever measurement differences could plausibly affect the outcome. Observations must meet a minimum data availability of 70% of expected values within the analysis window (14–90 days preceding the assessment), and those below the applicable threshold are reported rather than imputed.

#### Glucose metrics calculation

Both clinically established (including mean glucose, SD, coefficient of variation, time-in-range categories, time-in-tight-range) and advanced CGM metrics (including Continuous Overall Net Glycemic Action, Average Daily Risk Range, Glucose Fluctuation Moment Index)^17 19^ will be calculated according to previously published definitions (online supplemental table 2).

### Neurophysiology assessment

#### Nerve conduction studies

##### Assessment protocol

Four standardised peripheral nerves are evaluated annually: peroneal, tibial and median motor and median sensory nerves. The inclusion of motor and sensory components provides a comprehensive assessment, as sensory axons (which include small fibre components) outnumber motor axons by at least 9-to-1^39^ in peripheral nerves. This selection targets nerves with demonstrated sensitivity to diabetic neuropathy while ensuring reliable measurement across diverse paediatric developmental stages. The peroneal nerve receives particular attention given its pronounced vulnerability to early diabetic neuropathy.^9^

##### Standardised protocol

All measurements follow established paediatric neurophysiology protocols with strict environmental standardisation (room temperature-controlled at 21°C–23°C, measurement of skin temperature, standardised electrode placement, left extremity assessment unless contraindicated by injury or other factors requiring right-side assessment).^40^

Motor and sensory NCV and distal motor latency are the primary biomarkers. Secondary biomarkers include metrics for the dispersion of the compound muscle and sensory nerve action potential (CMAP) and the total electric energy required to achieve the maximum CMAP amplitude.^41^

##### Paediatric-specific adaptations

To account for the established relationship between body height and NCV, we calculate height-adjusted difference values (dNCV=measuredNCV-expectedNCV; additional information in the supplement section *Height adjusted NCV (dNCV*)). Expected values are derived from adjustment models based on our healthy control group,^9^ according to previously published models by Hyllienmark *et al*^7 42 43^ and can be found in online supplemental table 3. This approach distinguishes diabetes-related impairment from developmental and environmental confounders. Negative dNCV values indicate performance below normative expectations and vice versa. A differentiated analysis on the relationship between peroneal NCV and height, age and other influencing factors has been conducted.^9^ The adjustment models were fitted on control participants aged 7 to 21 years, following existing literature.^7 42 43^ dNCV is reported only for participants within this age range. The relevance of temperature and similar analyses for the other three nerves are still ongoing.

### High-resolution ultrasound

#### Structural assessment

High-resolution ultrasound provides direct visualisation of nerve structure. High-frequency probes (Canon Aplio i800 with i22LH8 probes) are used to assess structural nerve biomarkers including CSA and circumference of the median nerve.

#### Standardised protocol

Median nerve ultrasound is performed at three standardised anatomical sites on the left arm (or right arm if contraindicated) in the fully supinated position: wrist (L1), mid-forearm (L2) and distal upper arm (L3).^21 44 45^ CSA at L2 serves as the primary outcome measure due to its sensitivity for early neuropathic changes.^46^

Transverse images are captured at each location and stored for offline analysis.^21^ Trained operators manually trace the nerve border (hyperechoic rim between nerve tissue and surrounding connective tissue) to measure CSA and circumference. This retrospective measurement approach minimises patient discomfort and motion artefacts.

Additional metrics, including three-dimensional median nerve reconstruction and median nerve tortuosity (analogous to corneal nerve assessments in diabetic neuropathy),^47 48^ will be incorporated as methods become validated for ultrasound-based peripheral nerve assessment.^49^

#### Paediatric-specific adaptations

Median CSA is strongly correlated with body size^50^ and measurements are adjusted using BSA calculations (using the Du Bois formula^31^) to account for normal growth-related changes in nerve size throughout childhood and adolescence.^51^

### Laboratory measurements

All laboratory measurements are taken during the annual assessment, except for HbA1c and random capillary blood glucose, which are measured quarterly as shown in table 1.

HbA1c is measured using the Abbott AFINION 2 analyser, providing long-term data that complements CGM assessments. Random C-peptide is measured by chemiluminescence immunoassay (DiaSorin Liaison XL) to assess residual beta-cell function, which is particularly informative for the heterogeneous progression of beta-cell loss after diagnosis. Values below the assay’s limit of quantification (10 pmol/L) are recorded as zero. The ‘gold standard’ mixed meal tolerance test is not feasible in routine paediatric care and prandial timing is not recorded, so values reflect varying prandial states. Nevertheless, random C-peptide correlates with stimulated C-peptide and has been shown to predict diabetes complications.^22 52^ Capillary blood glucose is measured within ±1 hour of the neurophysiological assessment, so C-peptide is interpreted alongside the concurrent glucose concentration, including as a C-peptide-to-glucose ratio. Analyses of residual beta-cell function are accordingly exploratory.

Annual visits include comprehensive lipid profiles for metabolic risk assessment, thyroid and coeliac disease for diabetic comorbidities screening, and the albumin-to-creatinine ratio in the first voided morning urine for diabetic complications (table 1).

Electrolyte concentrations (sodium, potassium, chloride, magnesium and ionised calcium) are measured during the NCS appointment as important covariates for NCV.

## Patient and public involvement

The study’s care-embedded design ensures research questions address clinical concerns from routine paediatric diabetes practice. The focus on glucose variability and early neuropathy detection arose from family concerns about CGM data interpretation and long-term complication risk expressed during clinic visits.

Families have been involved since study inception through presentations at public events and media engagement.^53^ We continuously collect feedback regarding participation burden and assessment preferences during routine clinical encounters.

Individual results are discussed with patients and families after each annual examination. Aggregate findings are shared annually at parent evenings and youth diabetes events, and through presentations at diabetes patient and parent association meetings and diabetes centre events.

## Patient characteristics

Table 3 includes baseline demographic, anthropometric and neurophysiological characteristics of the KIND cohort and the healthy control group.

**Table 3:** Baseline characteristics of the control group and the KIND cohort

	Control group (n=123)			KIND cohort (n=179)		
	Median	(Q1; Q3)	n	Median	(Q1; Q3)	n
Demographics and anthropometrics						
Sex*						
Female (%)	56.1		69	49.2		88
Male (%)	43.9		54	50.8		91
Age (year)	10.8	(7.3; 14.6)	123	11.3	(7.6; 14.2)	179
Height (cm)	149.6	(124.5; 162.2)	110	148.8	(128.2; 164.1)	178
Height (Z-score)	0.4	(−0.3; 1.1)	110	0.5	(−0.1; 1.1)	177
Weight (kg)	43.6	(22.7; 55.3)	113	39.8	(25.0; 56.9)	177
Weight (Z-score)	0.2	(−0.5; 1.0)	111	0.3	(−0.3; 0.9)	175
BMI (kg/m²)	18.6	(15.6; 21.5)	109	17.9	(15.6; 20.9)	176
BMI (Z-score)	0.1	(−0.7; 0.9)	109	0.3	(−0.6; 0.9)	175
BSA (m^2^)	1.3	(0.9; 1.6)	109	1.3	(0.9; 1.6)	176
NCS						
Temperature (°C)	32.2	(31.4; 32.8)	83	31.8	(31.1; 32.3)	174
NCV peroneus (m/s)	51.0	(49.0; 54.0)	103	48.0	(46.0; 51.0)	178
dNCV peroneus (m/s)†	0.2	(−2.2; 2.3)	85	−3.1	(−5.9; −1.1)	149
NCV tibialis (m/s)	51.0	(48.0; 53.0)	89	47.0	(44.0; 51.0)	177
dNCV tibialis (m/s)†	−0.4	(−2.4; 2.0)	72	−3.5	(−6.2; −0.5)	149
NCV medianus motor (m/s)	58.0	(54.5; 60.5)	103	56.0	(53.0; 58.0)	179
dNCV medianus motor (m/s)†	−0.2	(−3.0; 2.4)	83	−2.0	(−4.1; 0.1)	150
NCV medianus sensory (m/s)	55.0	(51.0; 61.0)	97	54.0	(50.0; 57.0)	179
dNCV medianus sensory (m/s)†	−1.6	(−4.3; 3.7)	79	−2.6	(−6.9; −0.1)	150
Ultrasound						
L1 area (mm²)	7.2	(5.5; 9.8)	82	6.3	(5.0; 7.7)	160
L1 circumference (mm)	11.3	(9.9; 12.8)	78	11.2	(10.0; 12.9)	160
L2 area (mm²)	5.8	(4.5; 8.1)	81	5.8	(4.7; 7.2)	165
L2 circumference (mm)	9.0	(8.1; 10.0)	78	9.6	(8.5; 10.6)	165
L3 area (mm²)	6.8	(5.2; 8.1)	38	7.0	(5.2; 8.9)	113
L3 circumference (mm)	10.2	(9.5; 11.5)	36	10.7	(9.4; 12.4)	113

All values are median and the first and third quartiles, unless otherwise specified; n for the number of patients.

* Overall percentage, not median.

† dNCV is reported only for participants aged 7–21 years, the age range over which the adjustment models were fitted, so n is lower than for the corresponding measured velocities; see supplement section height adjusted NCV (dNCV).

BMI, Body Mass Index; BSA, Body Surface Area; dNCV, Height-adjusted Nerve Conduction Velocity; KIND, KINder mit Diabetes; NCS, Nerve Conduction Studies.

## Findings to date

Parts of this cohort have been previously published and presented at international conferences. As the cohort is continuously growing, patient numbers, characteristics and inclusion/exclusion criteria may differ between analyses. Detailed information is available in the cited reports.

### Cross-sectional neurophysiological findings

Cross-sectional NCS comparing 53 children with T1D (age 5.9–23.8 years) to 50 healthy controls (age 5.3–18.9 years) revealed significantly slower NCV across all four nerves examined (peroneal, tibial, median motor and median sensory), with the peroneal nerve most severely affected.^9^ After adjusting for height, peroneal dNCV correlated most strongly with glucose SD (r=−0.45, p=0.0085), more so than with the coefficient of variation (r=−0.26, p=0.031) and HbA1c (r=−0.27, p=0.049). During the first 5 years postdiagnosis, dNCV declines as diabetes duration progresses (r=−0.41, p=0.0041) independently of glycaemic control, hypothesised to reflect the end of the remission phase and a decrease of C-peptide secretion.

Figure 2 displays peroneal dNCV and HbA1c at baseline.

**Figure 2:**
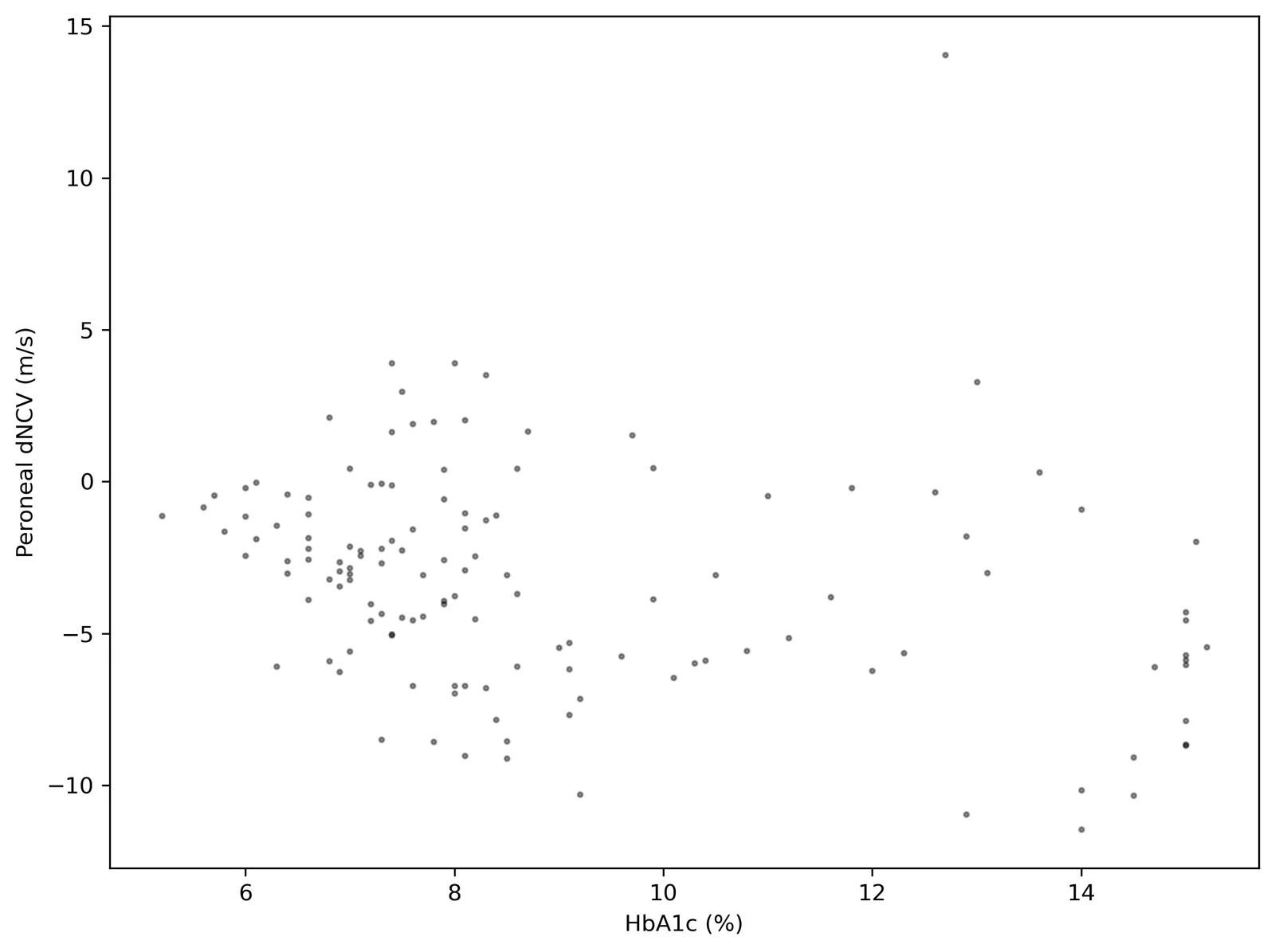
Peroneal dNCV and HbA1c at baseline. dNCV, Height-adjusted Nerve Conduction Velocity.

### Structural nerve changes detected by ultrasound

Complementary structural assessment, namely measurement of the CSA of the median nerve in the middle of the forearm using high-resolution ultrasound, demonstrated median nerve enlargement at mid-forearm in children with T1D, particularly those with HbA1c >9%.^46^ After adjusting for BSA, the mean CSA/BSA increased by 0.217 mm² per 1% HbA1c (p=0.004), with this association present even in children with diabetes duration <2 years, despite normal clinical examinations.

### Longitudinal associations and potential reversibility

Whereas the analyses above describe single time points, serial assessments allow change within individuals to be examined. A previous longitudinal analysis of 45 children with serial NCS (median interval 17.9 (16.1; 25.8) months) demonstrated that changes in HbA1c (${HbA1c}_{t1}-{HbA1c}_{t0}$) were significantly and negatively associated with changes in peroneal dNCV (${dNCV}_{t1}-{dNCV}_{t0}$; β=−0.59, p=0.014). In most patients, dNCV decreased with increasing HbA1c. Other patients showed improvements in dNCV with decreasing HbA1c, suggesting potential reversibility of early functional nerve impairment with improved glycaemic control.^54^

Figure 3 shows the evolution of peroneal dNCV across diabetes duration. Some patients exhibit a successive degradation of peroneal dNCV while others appear to improve over time. There are also multiple instances of an initial drop followed by a rebound in dNCV. One female patient, as an example, is exhibiting normal to very high dNCV across all four nerves (peroneal 14.05 m/s; tibial 0.52 m/s; median motor 0.26 m/s; median sensory 8.38 m/s) despite high HbA1c measured 6 days after diagnosis (age=14.2 years; HbA1c=12.7%; C-peptide=374 pmol/L; pH=7.38). These observations raise further questions, and we hope to be able to provide new insights in the course of this cohort study.

**Figure 3:**
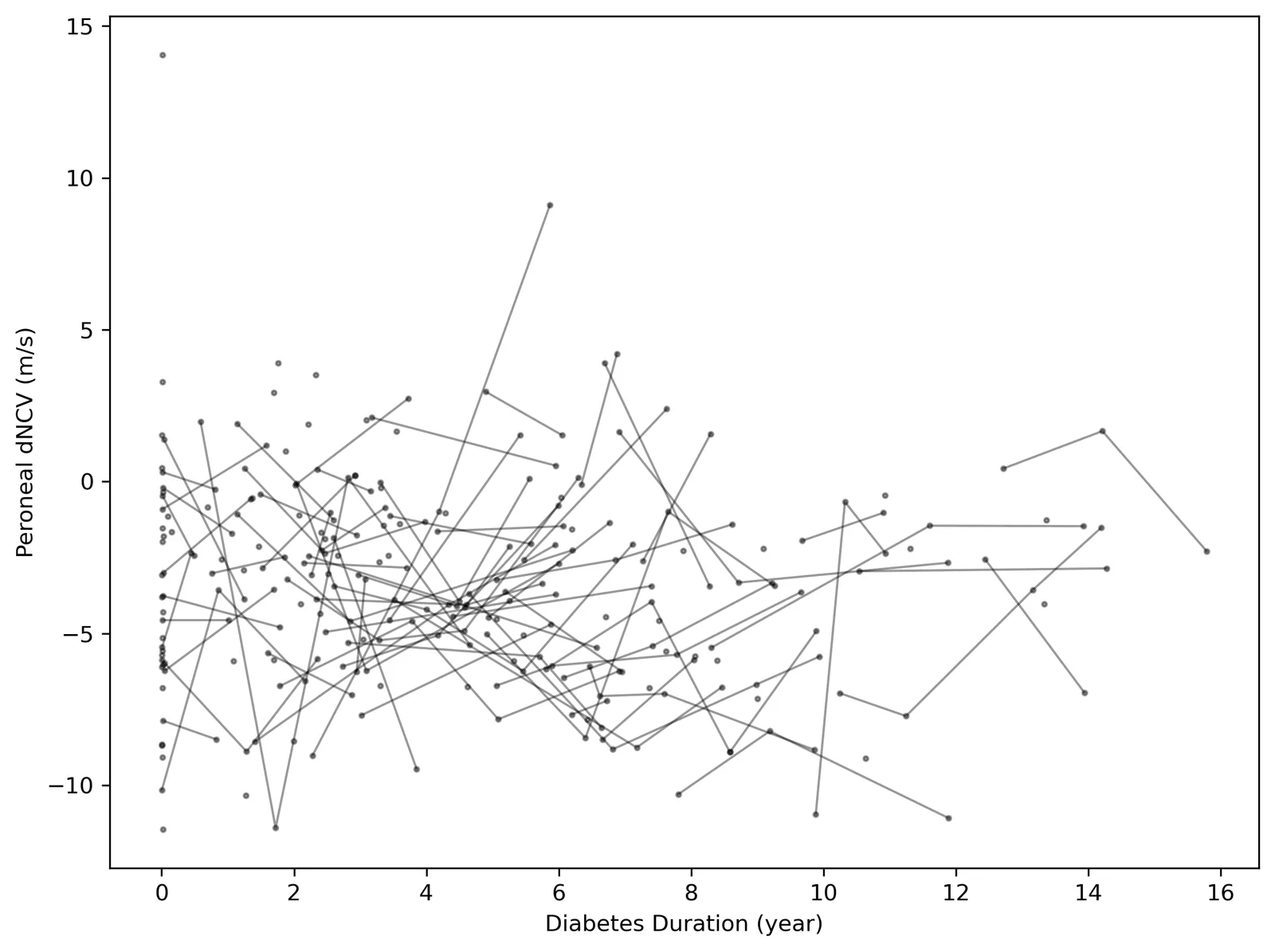
Peroneal dNCV across diabetes duration. Connected dots represent observations for the same patient. dNCV, Height-adjusted Nerve Conduction Velocity.

### Cohort follow-up to date

Of the 179 participants enrolled to date, 80 have completed 1 annual assessment, 64 have completed 2, 22 have completed 3, 12 have completed 4 and 1 has completed 5.

## Strengths and limitations

### Strengths

The care-embedded longitudinal design of the KIND cohort with continuous enrolment from T1D diagnosis minimises participant burden, maximises retention and captures neuropathy from its earliest clinically unapparent stages through adolescence, covering the full spectrum of diabetes duration and developmental stages. The study employs comprehensive non-invasive neurophysiological assessment combining functional (NCS across four nerves) and structural (high-resolution ultrasound) measures, with development-adjusted analyses (dNCV, BSA-adjusted CSA) that isolate diabetes-related impairment from normal growth using healthy control reference values. The longitudinal design with detailed glycaemic characterisation through quarterly HbA1c and continuous CGM data access enables direct comparison of established and advanced GV metrics (including GFMI and other complex indices) as predictors of neurophysiological outcomes over time, complemented by C-peptide assessment to investigate residual beta-cell function effects.

## Limitations

This regional two-centre study has limited geographic diversity that may affect generalisability. While the sample size is substantial for paediatric diabetic neuropathy research, the heterogeneous nature of the paediatric population (wide age range, developmental stages and diabetes duration) combined with multiple influencing factors limits statistical power for various analyses. CGM device heterogeneity across multiple manufacturers and models with different measurement characteristics complicates metric standardisation and comparability. Additionally, the control cohort comprises children presenting with unilateral injuries rather than healthy volunteers. NCSs are uncomfortable and not ethically justifiable in healthy children without a clinical indication, so a purely healthy reference group was not attainable. Conditions affecting peripheral nerve function are excluded, measurements are taken from the unaffected contralateral side, and the resulting reference values agree with published paediatric norms, but residual differences from the general paediatric population cannot be excluded.

Random C-peptide sampling without recorded prandial timing and incomplete CGM coverage among recently diagnosed participants further constrain the analyses depending on these variables.

## Supplementary material

10.1136/bmjopen-2026-116918 online supplemental file 1

## Data Availability

Data are available on reasonable request.
