# Supplementary material for: Longitudinal assessment of glycaemic management and neurophysiological outcomes in paediatric type 1 diabetes in Switzerland: the KIND cohort profile
Source: BMJ Open. 2026 Nov 11;16(11):e116918. doi: 10.1136/bmjopen-2026-116918 (PMC13756328; doi:10.1136/bmjopen-2026-116918)
Supplement: Supplementary file 1 [file bmjopen-2026-116918.reviewer_comments.pdf]

PEER REVIEW HISTORY

BMJ Open publishes all reviews undertaken for accepted manuscripts. Reviewers are asked to complete a checklist review form and are provided with free text boxes to elaborate on their assessment. These free text comments are reproduced below.

ARTICLE DETAILS

Title (Provisional)

The KIND cohort profile: longitudinal assessment of glycaemic management and neurophysiological outcomes in paediatric type 1 diabetes in Switzerland

Authors

Gruener, Marc Robin; West, Erin; Muhitira, Ute; Heldt, Katrin; Oberhauser, Sarah; l'Allemand-Jander, Dagmar; Broser, Philip Julian; KIND Study Group, on behalf of

---

VERSION 1 - REVIEW

**Reviewer**	**1**
**Name**	**Wahid, Abdul**
**Affiliation**	**Quaid-i-Azam University, Department of Pharmacy**
**Date**	**06-Mar-2026**
**COI**	**None**

• page 10, lines 40-42) lacks detail about exclusion criteria for controls (e.g., systemic conditions, medications affecting nerves). Add explicit inclusion/exclusion criteria for controls.

• The description of CGM device heterogeneity and harmonization challenges (pages 13-14) is thorough but lacks a clear, pre-specified statistical plan for addressing these issues. The statement "statistical model specifications may include device manufacturer and model as a covariate" is vague.

• Table 2 and 3 present comprehensive baseline data, but several important variables are missing or incompletely reported:

• The "Findings to date" section (page 20) presents important results but could be better organized with clear subheadings and appropriate citations to published work. The narrative jumps between cross-sectional, structural ultrasound, and longitudinal findings without clear transitions.

Restructure with subheadings:

"Cross-sectional neurophysiological findings"

"Structural nerve changes detected by ultrasound"

"Longitudinal associations and potential reversibility"

**Reviewer**	**2**
**Name**	**Fawwad, Asher**
**Affiliation**	**Baqai Institute of Diabetology and Endocrinology, Baqai Medical University, Research**
**Date**	**03-Aug-2026**
**COI**	**None**

No

**Reviewer**	**3**
**Name**	**Yokomichi, Hiroshi**
**Affiliation**	**University of Yamanashi, Department of Health Sciences**
**Date**	**23-Aug-2026**
**COI**	**None**

I would like to commend the authors for their tremendous efforts in establishing this valuable cohort study. This protocol holds significant potential to advance the current International Society for Pediatric and Adolescent Diabetes (ISPAD) guidelines. My primary comments and queries are outlined below:

1. Appropriateness of the control group

The control group comprises children presenting with various medical conditions/injuries rather than completely healthy volunteers. The clinical heterogeneity among these patients and the absence of a purely healthy control cohort represent notable limitations. I have some concerns regarding whether this group can serve as an adequately robust normative reference to evaluate neurophysiological observations in paediatric type 1 diabetes accurately. Nevertheless, I fully recognise the valuable potential of this study to advance future clinical guidelines.

2. CGM device heterogeneity and data handling

Given the differences across CGM manufacturers and device models, how do the authors plan to harmonise discrepancies in sampling intervals (e.g. 1-, 5-, and 15-minute recordings) and handle missing data in subsequent longitudinal analyses?

3. Measurement and utility of random C-peptide

C-peptide levels were measured on a random basis and will inevitably fluctuate depending on fasting or postprandial status. With sampling intervals and prandial timing varying across participants, how do the authors intend to account for or standardise these values in their statistical models? Furthermore, is this measurement strictly indispensable to achieving the core objectives of the study?

Overall, I consider this an important investigation with the potential to substantially advance clinical management in paediatric diabetes, and I eagerly look forward to seeing the future findings from this cohort.

VERSION 1 - AUTHOR RESPONSE

Reviewer 1 (Dr Abdul Wahid, Quaid-i-Azam University)

1. Page 10, lines 40–42 lacks detail about exclusion criteria for controls (e.g. systemic conditions, medications affecting nerves). Add explicit inclusion/exclusion criteria for controls.

We agree and have restructured the eligibility section. Criteria are now presented at two levels: general criteria applying to both arms (control & T1D, followed by arm-specific criteria.

General criteria are an age of 21 years or younger at enrolment, written informed consent from the participant and/or their legal guardians, and the absence of acute illness and any condition known to affect peripheral nerve function or structure. The latter comprises premature birth (< 32 gestational weeks), a personal or family history of inherited neuropathy or other hereditary neurological disease, known acquired polyneuropathy of any cause, current or previous treatment with neurotoxic agents including chemotherapy, vitamin B12 deficiency, untreated hypothyroidism, chronic kidney disease, and chronic inflammatory or rheumatological disease.

Arm-specific criteria apply in addition. Participants in the T1D arm require a diagnosis confirmed according to ISPAD clinical and laboratory criteria. Participants in the control arm require a unilateral upper-limb injury with a clinically unaffected contralateral side and are further excluded in the presence of diabetes or impaired glucose tolerance, or of suspected injury to the brachial plexus or to the nerves of the reference side.

We have also stated how eligibility was ascertained (from the clinical history taken at the neurophysiological appointment and from review of the clinical record) and that consecutively presenting patients meeting these criteria are invited to participate. The separate ethics approval covering the control arm (RAPS 2024-00672) is now cited alongside

the approval for the diabetes cohort.

2. The description of CGM device heterogeneity and harmonization challenges is thorough but lacks a clear, pre-specified statistical plan. The statement "statistical model specifications may include device manufacturer and model as a covariate" is vague.

We accept that the sentence quoted was too vague and have replaced it.

The CGM section now states the pre-processing applied in our analyses conducted to date: all recordings are resampled to a common 15-minute grid by forward-fill with a limit of one interval, and values outside the device-specific measurement range are treated as missing. It further states that device manufacturer and model are carried as covariates, or as the basis of sensitivity analyses restricted to a single device, wherever systematic measurement differences could plausibly affect the outcome, and that observations must meet a minimum data availability within the relevant window, typically 70 % of expected values, with observations below the applicable threshold reported alongside each analysis rather than imputed. We have also made explicit why a single fixed rule is not appropriate: velocity-based metrics such as MAG, GVP and the GFMI are considerably more sensitive to sampling frequency than mean glucose, standard deviation or time-in-range, so the appropriate temporal alignment, out-of-range coding and sufficiency threshold depend on which metric is under study.

We hope the reviewer will accept that a full pre-specified statistical analysis plan cannot be presented here. BMJ Open's instructions for the Cohort profile article type state explicitly that "Detailed statistical plans should not be reported." The cohort is described at protocol level, and the analyses that will draw on these data are at varying stages of development; committing the cohort to a single harmonization rule would misrepresent how these decisions are properly made. We have therefore specified the rules used so far and the principles governing future choices, which we believe addresses the substance of the comment within the constraints of the article type.

3. Table 2 and 3 present comprehensive baseline data, but several important variables are missing or incompletely reported.

The itemized list following this comment did not reach us, and we have addressed the comment based on the text received.

Table 3 now additionally reports pH and bicarbonate at diagnosis, and diabetic ketoacidosis at diagnosis classified according to ISPAD 2022 criteria, including severity. Both tables have been updated to the enlarged cohort, and all denominators within a given block are now the total number of participants in that arm, so that proportions are directly comparable across

rows. The number of participants contributing to each variable continues to be reported in a dedicated column, and the footnotes now explain the two situations in which n departs from the total: dNCV, which is reported only within the 7–21 year fitting range of the adjustment models, and DKA classification, for which the blood gas obtained at presentation was unavailable in 17 participants, reported as a Missing row in Table 3.

We have also added an explanation of the CGM coverage figures, which we expect would otherwise invite misinterpretation. The proportion of participants with adequate CGM data at baseline has fallen relative to the original submission even though the absolute number has risen. This reflects the continuous enrolment design: over 30% of the cohort is now enrolled within 3 months of diagnosis, before CGM therapy is established.

Variables describing insulin delivery method (multiple daily injections versus continuous subcutaneous insulin infusion) and device configuration are held in the clinical information system. Variables describing insulin delivery method (multiple daily injections versus continuous subcutaneous insulin infusion) and device configuration are recorded in the clinical information system and will be reported in the analyses addressing the fifth study objective.

4. The “Findings to date” section presents important results but could be better organized with clear subheadings and appropriate citations to published work. The narrative jumps between cross-sectional, structural ultrasound, and longitudinal findings without clear transitions. Restructure with subheadings.

We have adopted the reviewer’s suggestion in full. The section is now organized under the three subheadings proposed (“Cross-sectional neurophysiological findings”, “Structural nerve changes detected by ultrasound” and “Longitudinal associations and potential reversibility”) with an added fourth subheading, “Cohort follow-up to date”, covering the number of assessments completed. A transition has been added into the longitudinal subsection, and citations to the relevant publications are given at the head of each subsection. The follow-up figures have been updated: of the 179 participants enrolled, 80 have completed one annual assessment, 64 two, 22 three, 12 four, and one has completed five.

Reviewer 2 (Dr Asher Fawwad, Baqai Institute of Diabetology and Endocrinology)

We thank the reviewer for the assessment and note that no comments requiring a response were raised.

Reviewer 3 (Dr Hiroshi Yokomichi, University of Yamanashi)

We are grateful for the reviewer's generous assessment of the cohort and for three comments that go to the heart of the design.

1. Appropriateness of the control group. The control group comprises children presenting with various medical conditions/injuries rather than completely healthy volunteers. The clinical heterogeneity among these patients and the absence of a purely healthy control cohort represent notable limitations.

We share the reviewer's concern and have addressed it in three ways, while acknowledging that it cannot be resolved by design.

First, on feasibility: nerve conduction studies are uncomfortable and carry no clinical benefit for a healthy child. A study recruiting healthy children solely to undergo them would not, in our judgement and that of our ethics committee, be justifiable. A purely healthy paediatric reference group of this size was therefore not attainable, and we now say so explicitly rather than leaving it implicit.

Second, on heterogeneity: the reference group is not a general clinical population. It comprises children attending presenting with a unilateral upper-limb injury and a clinically unaffected contralateral side, and the exclusion criteria set out in response to Reviewer 1 (including acute illness, diabetes and impaired glucose tolerance, hereditary and acquired neuropathies, neurotoxic medication, and systemic conditions affecting nerve function) apply to this group as they do to the diabetes arm. Measurements are taken from the uninjured side, a within-patient design that has been used for this purpose previously.

Third, on empirical adequacy: the reference values derived from this group agree closely with published paediatric normative data. Supplementary Figure 1 now plots the height regressions fitted on our control group against the normative values of Hyllienmark et al. for all four nerves; the two are closely comparable across the height range covered by the cohort. The correspondence was also demonstrated in the supplement of Oberhauser et al. (Diabetes 2023), the study from which this reference group is drawn. The median nerve cross-sectional area data are similarly anchored in previously published paediatric reference values from our group. We have refitted the adjustment models on the enlarged control group for this manuscript, restricted to participants aged 7 to 21 years; the number contributing to each nerve is given in Supplementary Table 3.

We have nonetheless strengthened the limitation. The Limitations section now states plainly that the reference group comprises injured rather than healthy children, that a purely healthy group was not ethically attainable, and that residual differences between this group and the

general paediatric population cannot be excluded. A corresponding point has been added to the Strengths and limitations of this study box.

2. CGM device heterogeneity and data handling. How do the authors plan to harmonize discrepancies in sampling intervals (e.g. 1-, 5-, and 15-minute recordings) and handle missing data in subsequent longitudinal analyses?

The reviewer's question is answered in part in the supplement, which we accept was not sufficiently described in the main text. We have promoted the relevant content.

On sampling intervals: recordings are resampled to a common 15-minute grid by forward-fill with a limit of one interval, 15 minutes being the coarsest native resolution among the devices in use. In practice the realistic range is 5 to 15 minutes; 1-minute data are available only for a small subset. The choice of a common grid matters unevenly across metrics, and we now say so in the main text: mean glucose, standard deviation and time-in-range are largely robust to resolution, whereas velocity-based metrics (MAG, GVP and the GFMI) are sensitive to it, since they are computed from successive differences. For analyses centered on these metrics the resampling rule is therefore a substantive analytical decision rather than a preprocessing detail, and will be specified, with sensitivity analysis, in the analysis concerned.

On values outside the measurement range: To our knowledge, no consensus on the handling of these values exists in the literature and different approaches (e.g. coded as missing or clamped to the range limits) have a different impact on derived glucose metrics, especially variability ones. The final decision will be made on a case-by-case basis and documented in future original research manuscripts accordingly. Additionally, sensitivity analyses using alternative strategies are planned. Supplementary Table 4 reports the realised data availability and the proportion of values excluded as out of range in the subgroup meeting the 70 % threshold.

On missing data: Observations are required to meet a minimum data availability within the relevant window, set at 70 % of expected values in the analyses conducted so far, with a shorter-window fallback of 14 to 90 days where applicable. Observations not meeting the threshold are reported alongside each.

We would add one point of transparency prompted by this comment. The proportion of participants with adequate CGM coverage at baseline is lower in this revision than in the original submission, despite the absolute number having increased, because continuous enrolment means a growing share of the cohort is assessed at or shortly after diagnosis, before CGM therapy is established. This is now explained in the text accompanying Table 3.

3. Measurement and utility of random C-peptide. C-peptide levels were measured on a random basis and will inevitably fluctuate depending on fasting or postprandial status. How do the authors intend to account for or standardize these values? Is this measurement strictly indispensable?

The reviewer is correct, and we have revised the manuscript to state the limitation rather than to defend the measurement.

The “gold standard” mixed meal tolerance test is not feasible within routine paediatric diabetes care without imposing a burden inconsistent with the care-embedded design of the cohort, and we do not record the time since the last meal or the last insulin dose. Random C-peptide values therefore reflect varying prandial states, and this is now stated explicitly in the Laboratory Measurements section and in the Limitations.

One feature of the data partially mitigates this. Capillary blood glucose is measured within ±1 h of the neurophysiological assessment, so C-peptide can be interpreted alongside the concurrent glucose concentration, including as a C-peptide-to-glucose ratio. Values below the assay's lower limit of quantification are recorded as zero, which is now stated.

We have, however, accepted the implication of the reviewer’s question and now describe analyses of residual beta-cell function as exploratory rather than confirmatory. Nevertheless, random C-peptide correlates with stimulated C-peptide and has been shown to predict diabetes complications.

We thank the reviewers again for their time and for comments that have materially improved the manuscript.
