# Supplementary material for: Longitudinal assessment of glycaemic management and neurophysiological outcomes in paediatric type 1 diabetes in Switzerland: the KIND cohort profile
Source: BMJ Open. 2026 Nov 11;16(11):e116918. doi: 10.1136/bmjopen-2026-116918 (PMC13756328; doi:10.1136/bmjopen-2026-116918)
Supplement: Supplementary file 2 [file bmjopen-16-11-s001.docx]

# 1 Supplementary Materials

## 1.1 Comparison of different CGM Sensors

There are relevant differences in the characteristics of different CGM devices and manufacturers and derived metrics^1^.

Supplementary Table 1: Comparison of different CGM sensors
(MARD = Mean Absolute Relative Difference)

## 1.2 Advanced Glucose Variability Metrics

The following glucose variability metrics are briefly described in terms of their potential ability to predict slowing of NCV as early sign of subclinical diabetic peripheral neuropathy in children and adolescents with T1D.

The Continuous Overall Net Glycemic Action (CONGAn) measures intraday glycaemic variation through glucose differences at defined time intervals. Validated in paediatric Type 1 diabetes populations, this metric enables flexible examination of both short- and long-term glucose patterns.^2^

The Average Daily Risk Range (ADRR) accounts for asymmetric glucose variations through distinct risk functions for hypoglycaemia and hyperglycaemia. Logarithmic transformation enables balanced evaluation of extreme glycaemic events and overall risk.^3–5^

The Mean Absolute Glucose Change (MAG) captures the total magnitude of glucose fluctuations over time, distinguishing between profiles with comparable means or standard deviations but different control patterns. This metric proves particularly useful for detecting clinically relevant variations in intensive care environments.^6^

The Glycemic Variability Percentage (GVP) employs a geometric approach, comparing actual glucose trajectory lengths to theoretical minimum lengths. This method specifically identifies high-frequency and high-amplitude fluctuations, offering complementary insight into glucose stability.^7^

We introduce the Glucose Fluctuation Moment Index (GFMI), combining squared glucose change velocity with squared deviation from the 5 mmol/L target.^8^ The GFMI highlights substantial glucose shifts occurring distant from target range:

$$GFMI=\frac{1}{n}\sum_{i=1}^{n} {(g_{t=i+1}-g_{t=i})}^{2}*{(g_{t=i}-5)}^{2}$$

Supplementary Table 2: Characteristics of selected advanced glucose variability metrics (Reproduction of Gruener et al.^9^)

| Metric | Definition and Purpose | Formula | Key Clinical & Scientific Applications | Distinguishing Features |
| --- | --- | --- | --- | --- |
| Continuous Overall Net Glycemic Action (CONGAn)^2^ | Measures glycaemic variation by analysing differences between glucose readings at specified time intervals (n hours) | $CONGAn = \sqrt{\frac{\sum_{t=t_{1}}^{t_{k^{*}}} \left( D_{t}-\bar{D} \right)^{2}}{k^{*}-1}}$ $with D_{t}=GR_{t}-GR_{t-m}$ $and \bar{D} =\frac{\sum_{t=t_{1}}^{t_{k^{*}}} D_{t}}{k^{*}}$ $and k^{*}=n x 60min$ | Association with distal symmetric polyneuropathy in T1D children; Validation of synthetic CGM data; Assessment of physical activity impact on glycaemic variability ^10–15^ | Flexible time intervals (1h, 2h, 4h, 12h, 24h) for different clinical objectives; captures temporal glucose dynamics |
| Average Daily Risk Range (ADRR)^3–5^ | Quantifies combined risk of hypo- and hyperglycaemia through logarithmic transformation of glucose values | $ADRR = \frac{1}{M}\sum_{i=1}^{M} [LR^{i}+{HR}^{i}]$  $with {LR}^{i}=max[rl(x_{1}^{i}), ..., rl(x_{n}^{i})] and {HR}^{i}=max[rh(x_{1}^{i}), ..., rh(x_{n}^{i})] for day i; i= 1,2, ..., M$  $and rl\left( BG \right)= r\left( BG \right)if f\left( BG \right)<0 else 0$  $and rh(BG)= r(BG) if f(BG)>0 else 0$  $and r(BG) = f(BG) =1.509*[{ln(BG)}^{1.084}-5.381]$ | Evaluation of T1D and T2D treatment regimens; Mortality prediction in burn ICU patients; Assessment of diabetes support systems^10,11,16–19^ | Addresses skewness in CGM data through logarithmic transformation; combines both high and low glucose risks |
| Mean Absolute Glucose Change (MAG)^6^ | Measures the sum of absolute glucose changes over time, designed to capture clinically relevant glucose variations | $MAG=\frac{\left\vert\Delta BG \right\vert}{\Delta Time}$ | Prediction of ICU mortality; Assessment of critically ill non-diabetic children; Classification of gastroparesis in T1D^6,20,21^ | Differentiates between clinically distinct glucose patterns that may have similar means/SDs; Particularly relevant for ICU settings |
| Glycemic Variability Percentage (GVP)^7^ | Compares actual glucose trace length to minimal possible length, emphasizing oscillating excursions | $GVP=(\frac{L}{L_{0}}-1)*100$  $with L=\sum_{i=1}^{n} \sqrt{\Delta x_{i}^{2}+\Delta y_{i}^{2}}$  $and L_{0}=\sum_{i=1}^{n} \Delta x_{i}$ | Differentiation between T2D and impaired glucose tolerance; Prediction of nocturnal hypoglycaemia; Evaluation of automated insulin delivery^22–25^ | Penalizes high-frequency and high-amplitude oscillations; Geometric approach to variability assessment |
| Glucose Fluctuation Moment Index (GFMI)^8^ | Integrates velocity of glucose change weighted by distance from glycaemic target | $GFMI=\frac{1}{n}\sum_{i=1}^{n} {(g_{t=i+1}-g_{t=i})}^{2}*{(g_{t=i}-5)}^{2}$ | Under investigation for association with nerve vitality^8^ | Uniquely weights changes by distance from target; Emphasizes substantial changes in glucose concentration away from target range |

## 1.3 Height adjusted nerve conduction velocity (dNCV)

As outlined in section *Nerve Conduction Studies* and previously described in Oberhauser et al.^26^, we define *height adjusted Nerve Conduction Velocity* (*dNCV)* as the difference between the measured NCV and the NCV expected for a child of a certain height $h$ in cm ($dNCV= {NCV}_{measured}- {NCV(h)}_{expected}$). The expected NCV is determined by fitting a linear regression model ${NCV}_{expected} \sim Height \left( cm \right)$ to the data obtained from our healthy control group, adjusted according to Hyllienmark et al.^27–29^. The resulting control regressions are shown against the normative values published by Hyllienmark et al. in Supplementary Figure 1; the two are closely comparable across the height range covered by the cohort, supporting the use of this reference group for developmental adjustment. The relevance of age and other influencing factors (e.g. temperature) in addition to height is currently being evaluated for the peroneal, tibial as well as median motor and sensory nerves in the T1D cohort.

Supplementary Table 3: Linear regression models for the healthy control group.
Models were fitted on control participants aged 7 to 21 years, following Hyllienmark et al.; dNCV is calculated only for participants within this age range.

| **Nerve** | **Intercept** | **β Height** | **P>\|t\|** | **95% CI** | **n** |
| --- | --- | --- | --- | --- | --- |
| Peroneus (m/s) | 63.5348 | -0.0772 | 0.001 | [-0.121; -0.034] | 66 |
| Tibialis (m/s) | 61.1123 | -0.0652 | 0.037 | [-0.126; -0.004] | 56 |
| Medianus motor (m/s) | 50.4485 | 0.0509 | 0.071 | [-0.005; 0.106] | 67 |
| Medianus sensory (m/s) | 50.5689 | 0.0433 | 0.309 | [-0.041; 0.128] | 64 |

Supplementary Figure 1: NCV compared to height in healthy controls

## 1.4 Subgroup CGM characteristics of the KIND Cohort

Data were filtered to include, for each KIND cohort participant, the first assessment at which at least 70 % of the expected CGM values were available for the 90 days preceding the NCS appointment. This is not necessarily the participant’s baseline assessment, so the number of participants reported here exceeds the number with adequate CGM coverage at baseline given in Table 3 of the main manuscript. Data was resampled from 1, 5, or 15min, respectively, to 15min periods (via forward-fill with a limit of 1) where 100% data availability equates to 8’640 CGM ($90 days * 24 h/day * 60 min/h / 15min = 8’640$) values post-resampling. Values out of measurement range were marked as missing. After filtering, 85 participants remained.

Supplementary Table 4: Subgroup characteristics of the 85 participants at their first assessment with ≥70 % CGM data availability
*100% equals 8’640 CGM values after resampling to 15 min

|  | **Median** | **[Q1; Q3]** |
| --- | --- | --- |
| Data Availability (%)* | 95.76 | [88.91; 97.99] |
| CGM values excluded “low” (%)* | 0.08 | [0.00; 0.38] |
| CGM values excluded “high” (%)* | 1.00 | [0.27; 5.67] |
| Mean Glucose (mmol/L) | 9.08 | [8.54; 9.74] |
| Median Glucose (mmol/L) | 8.40 | [7.80; 9.15] |
| Glucose Standard Deviation | 3.70 | [3.17; 4.14] |
| Glucose CV (%) | 39.23 | [37.12; 42.77] |
| TIR | | |
| very low (%) | 0.41 | [0.18; 0.75] |
| low (%) | 1.98 | [1.16; 3.52] |
| normal (%) | 61.16 | [54.41; 70.85] |
| high (%) | 21.99 | [19.82; 24.73] |
| very high (%) | 11.97 | [6.46; 17.96] |
| ADRR | 46.22 | [39.59; 54.18] |
| CONGA24 | 4.58 | [4.10; 5.34] |
| GVP | 29.39 | [20.38; 36.18] |
| MAG | 3.23 | [2.69; 3.66] |
| GFMI | 49.42 | [31.75; 77.94] |

# 2 References

1	Freckmann G, Wehrstedt S, Eichenlaub M, *et al.* A Comparative Analysis of Glycemic Metrics Derived From Three Continuous Glucose Monitoring Systems. *Diabetes Care* 2025; **48**: 1213–7.

2	McDonnell CM, Donath SM, Vidmar SI, Werther GA, Cameron FJ. A Novel Approach to Continuous Glucose Analysis Utilizing Glycemic Variation. *Diabetes Technol Ther* 2005; **7**: 253–63.

3	Kovatchev BP, Cox DJ, Gonder-Frederick LA, Clarke W. Symmetrization of the blood glucose measurement scale and its applications. *DIABETES CARE* 1997; **20**.

4	Kovatchev BP, Clarke WL, Breton M, Brayman K, McCall A. Quantifying Temporal Glucose Variability in Diabetes via Continuous Glucose Monitoring: Mathematical Methods and Clinical Application. *Diabetes Technol Ther* 2005; **7**: 849–62.

5	Kovatchev BP, Otto E, Cox D, Gonder-Frederick L, Clarke W. Evaluation of a New Measure of Blood Glucose Variability in Diabetes. *Diabetes Care* 2006; **29**: 2433–8.

6	Hermanides J, Vriesendorp TM, Bosman RJ, Zandstra DF, Hoekstra JB, DeVries JH. Glucose variability is associated with intensive care unit mortality. *Crit Care Med* 2010; **38**: 838–42.

7	Peyser TA, Balo AK, Buckingham BA, Hirsch IB, Garcia A. Glycemic Variability Percentage: A Novel Method for Assessing Glycemic Variability from Continuous Glucose Monitor Data. *Diabetes Technol Ther* 2018; **20**: 6–16.

8	Gruener M-R, West E, l’Allemand D, *et al.* A Novel Digital Biomarker on the Impact of Glucose Fluctuations on Nerve Conduction Velocity in Pediatric T1D. In: Neuropediatrics. Georg Thieme Verlag KG, 2024: A-157.

9	Gruener MR, West E, Muhitira U, *et al.* Assessing Glucose Variability Metrics: Nerve Conduction Velocity in Children and Adolescents with Type 1 Diabetes. 2025; : 2025.03.25.25324553.

10	Rodbard D, Bailey T, Jovanovic L, Zisser H, Kaplan R, Garg SK. Improved quality of glycemic control and reduced glycemic variability with use of continuous glucose monitoring. *Diabetes Technol Ther* 2009; **11**: 717–23.

11	Oe Y, Nomoto H, Nakamura A, *et al.* Switching from Insulin Degludec plus Dipeptidyl Peptidase-4 Inhibitor to Insulin Degludec/Liraglutide Improves Glycemic Variability in Patients with Type 2 Diabetes: A Preliminary Prospective Observation Study. *J Diabetes Res* 2022; **2022**: 5603864.

12	Christensen MMB, Hommel EE, Jørgensen ME, Fleischer J, Hansen CS. Glycemic Variability and Diabetic Neuropathy in Young Adults With Type 1 Diabetes. *Front Endocrinol* 2020; **11**. DOI:10.3389/fendo.2020.00644.

13	Cichosz SL, Xylander AAP. A Conditional Generative Adversarial Network for Synthesis of Continuous Glucose Monitoring Signals. *J Diabetes Sci Technol* 2021; **16**: 1220.

14	Sparks JR, Sarzynski MA, Davis JM, Grandjean PW, Wang X. Cross-Sectional and Individual Relationships between Physical Activity and Glycemic Variability. *Transl J Am Coll Sports Med* 2022; **7**: 1.

15	Sparks JR, Sarzynski MA, Davis JM, Grandjean PW, Wang X. Alterations in Glycemic Variability, Vascular Health, and Oxidative Stress following a 12-Week Aerobic Exercise Intervention-A Pilot Study. *Int J Exerc Sci* 2021; **14**: 1334.

16	Sandig D, Grimsmann J, Reinauer C, *et al.* Continuous Glucose Monitoring in Adults with Type 1 Diabetes: Real-World Data from the German/Austrian Prospective Diabetes Follow-Up Registry. *Diabetes Technol Ther* 2020; **22**: 602–12.

17	Bisio A, Anderson S, Norlander L, *et al.* Impact of a Novel Diabetes Support System on a Cohort of Individuals With Type 1 Diabetes Treated With Multiple Daily Injections: A Multicenter Randomized Study. *Diabetes Care* 2021; **45**: 186.

18	Farhy LS, Ortiz EA, Kovatchev BP, Mora AG, Wolf SE, Wade CE. Average Daily Risk Range as a Measure of Glycemic Risk Is Associated with Mortality in the Intensive Care Unit: A Retrospective Study in a Burn Intensive Care Unit. *J Diabetes Sci Technol* 2011; **5**: 1087.

19	Lipska KJ, Venkitachalam L, Gosch K, *et al.* Glucose Variability and Mortality in Patients Hospitalized With Acute Myocardial Infarction. *Circ Cardiovasc Qual Outcomes* 2012; **5**: 550–7.

20	Dong M, Liu W, Luo Y, *et al.* Glycemic Variability Is Independently Associated With Poor Prognosis in Five Pediatric ICU Centers in Southwest China. *Front Nutr* 2022; **9**. DOI:10.3389/fnut.2022.757982.

21	Cichosz SL, Hejlesen O. Classification of Gastroparesis from Glycemic Variability in Type 1 Diabetes: A Proof-of-Concept Study. *J Diabetes Sci Technol* 2021; **16**: 1190.

22	Longato E, Acciaroli G, Facchinetti A, Maran A, Sparacino G. Simple Linear Support Vector Machine Classifier Can Distinguish Impaired Glucose Tolerance Versus Type 2 Diabetes Using a Reduced Set of CGM-Based Glycemic Variability Indices. *J Diabetes Sci Technol* 2019; **14**: 297–302.

23	Jensen MH, Dethlefsen C, Vestergaard P, Hejlesen O. Prediction of Nocturnal Hypoglycemia From Continuous Glucose Monitoring Data in People With Type 1 Diabetes: A Proof-of-Concept Study. *J Diabetes Sci Technol* 2019; **14**: 250–6.

24	Kaur RJ, Deshpande S, Pinsker JE, *et al.* Outpatient Randomized Crossover Automated Insulin Delivery Versus Conventional Therapy with Induced Stress Challenges. *Diabetes Technol Ther* 2022; **24**: 338–49.

25	Deshpande S, Pinsker JE, Church MM, *et al.* Randomized Crossover Comparison of Automated Insulin Delivery Versus Conventional Therapy Using an Unlocked Smartphone with Scheduled Pasta and Rice Meal Challenges in the Outpatient Setting. *Diabetes Technol Ther* 2020; **22**: 865–74.

26	Oberhauser SS, l’Allemand D, Willems EP, *et al.* Slowing of Peripheral Nerve Conduction Velocity in Children and Adolescents With Type 1 Diabetes Is Predicted by Glucose Fluctuations. *Diabetes* 2023; **72**: 1835–40.

27	Hyllienmark L, Brismar T, Ludvigsson J. Subclinical nerve dysfunction in children and adolescents with IDDM. *Diabetologia* 1995; **38**: 685–92.

28	Hyllienmark L, Ludvigsson J, Brismar T. Normal values of nerve conduction in children and adolescents. *Electroencephalogr Clin Neurophysiol* 1995; **97**: 208–14.

29	Hyllienmark L, Alstrand N, Jonsson B, Ludvigsson J, Cooray G, Wahlberg-Topp J. Early Electrophysiological Abnormalities and Clinical Neuropathy. *Diabetes Care* 2013; **36**: 3187–94.
